# Graphite-oxide hybrid multi-degree of freedom resonator metamaterial for broadband sound absorption

**DOI:** 10.1038/s41598-022-14415-3

**Published:** 2022-08-26

**Authors:** F. Bucciarelli, G. P. Malfense Fierro, M. Rapisarda, M. Meo

**Affiliations:** Department of Mechanical Engineering, University of Bath, Claverton Down, Bath, BA2 7AY UK

**Keywords:** Acoustics, Aerospace engineering

## Abstract

Low frequency broadband sound absorption for thin structures is still a great challenge. A new concept of a stackable hybrid resonator metamaterial is proposed which exhibits super broadband low-frequency sound absorption. The proposed metamaterial is based on micrometric scale thickness Graphene Oxide (GO) embedded in a stacked structure or used as external skin in a designed honeycomb (HC) structure. The stackable nature of the proposed structure allows the GO-HC cores to be embedded within micro-perforated panels (MPP) providing enhanced stiffness/strength to the structure and high absorption characteristics. We demonstrate how the exploitation of the GO elastic and mass properties result in multiple hybrid structural–acoustic resonances. These resonances are tailored to occur in a frequency range of interest by the theoretical calculation of the sound absorption coefficient. The theoretical model combines the mutual interaction between the structural dynamic of the GO foil and acoustic higher modes of the HC core cell as well as stacked MPP-HC/GO-HC cores. The result is a multi-degree of freedom hybrid resonator which provides subwavelength scale broadband sound absorption in low frequency range between 300 and 2500 Hz.

## Introduction

Acoustic metamaterials are engineered materials with periodic structure which have wide range of capabilities in sound wave manipulations, enabling phenomena such as acoustic cloaking^1^, sound insulation^2,3^ and sound absorption^4–7^. This new class of materials show unusual physical behaviours such as negative effective, modulus^8,9^, negative effective density^10,11^ and both the negative effects simultaneously^12^. Focusing on the sound absorption, the development of acoustic metamaterials are trying to meet the growing and challenging need for broadband sound absorption at low frequencies while maintaining sub-wavelength thickness. Porous absorbers need considerable thickness to maximize the absorption performance at low frequencies since the minimum thickness required is usually one order of magnitude smaller than the incident wavelength^13–15^. Based on localised resonances^10,16–18^ and monopolar/dipolar resonances^19,20^, several membrane-type metamaterials have been proposed in last few years which can fully absorb low frequency sound waves with a deep subwavelength. However, most of these devices behave as a single degree of freedom resonator, thus as the wavelength increases relative to the size of the absorber, the narrower the frequency band^21^. Metamaterials based on the space-coiling approach^22^ are suitable for full low frequency absorption. This class of metamaterial allow for the design of ultrathin structures and overcome the 1/4 wavelength (λ) limitation of resonators using coplanar coiled chambers^23–25^. The effective absorption frequency band of metamaterials can be increased by combining multiple single resonators or multiple Fabry–Perot channels in series or parallel^26,27^. An effective approach for reducing the size of the structure and ensuring broadband absorption is by combining the effect of a microperforated panel (MPP) with a coiled up Fabry–Perot channels^28^ or honeycomb structure^29^. In particular, Tang^30^ proposed a hybrid metamaterial where a MPP was combined with a perforated honeycomb-corrugation hybrid core achieving an absorption level over 50% in a frequency range between 29 and 1000 Hz with a structural thickness of 60 mm. Gao^31^ combined MPP with cavity, porous material (melamine polymer) and four lateral plates in order to assembly an optimized three units composite structure which allows broadband sound absorption from 200 Hz up 1.6 kHz with global thickness of 123.5 mm.

However, the problem of expanding the low frequency band of subwavelength metamaterial absorber remains relevant and crucial for acoustic engineering.

Recently, Graphene Oxide (GO). has attracted interest in acoustic engineering for sound absorption applications. Nine et al.^32^ used a melamine foam as structural support to create a lamella network of self-assembled GO micro-sheets obtained by dipping the support foam in an aqueous GO solution. This 26 mm GO lamella network increased the air-flow resistance and tortuosity consequently enhancing sound absorption coefficient resulting in 60% absorption over 800 Hz. Good sound absorption performance was achieved by Oh et al.^33,34^ who proposed a periodically self-aligned and hierarchically porous graphene-polyurethane foam. The 3D graphene microstructure into the open cell of existing polyurethane foam was achieved by hybrid physical–chemical crosslinking of graphene oxide liquid crystal-like structure. The result is a broadband absorption over 60% between 1000 and 6300 Hz. Recently, Lu et al.^35^ developed a 60 mm thickness bubbled graphene monolith, obtained via the freeze-casting of a GO dispersion, with a normalized absorption coefficient of 0.9 from about 60–6300 Hz.

In this report, a new class subwavelength hybrid metamaterial suitable for low frequencies broadband sound absorption by exploiting plate-like resonator properties of GO combined with a microperforated honeycomb core is proposed. Starting from designed and machined Honeycomb core structure (HC) with millimetre scale square unit cells; the micrometric thickness GO foil embedded on the HC core and the external skin is a millimetric Microperforated Panel (MPPHCGOHC). This defines a plate-type resonator metamaterial, where the GO foil is studied as a vibrating plate and the HC core unit cell represents the acoustic resonator chamber. This plate-type effect of the GO-HC structure is combined with the MPP absorber properties where the HC core unit cell is still the resonator chamber of the MPP absorber. We demonstrate how the proposed GO based metamaterials behave as a multi degree of freedom resonator, generating hybrid structural–acoustic resonances due to the mutual interaction of GO foil vibration resonances and higher acoustic resonance modes which contribute to the dissipation energy of the incoming sound wave.

As a results the proposed hybrid metamaterial provide a high broadband sound absorption in a frequency range 300–2500 Hz.

## Theoretical framework

**Figure 1:**
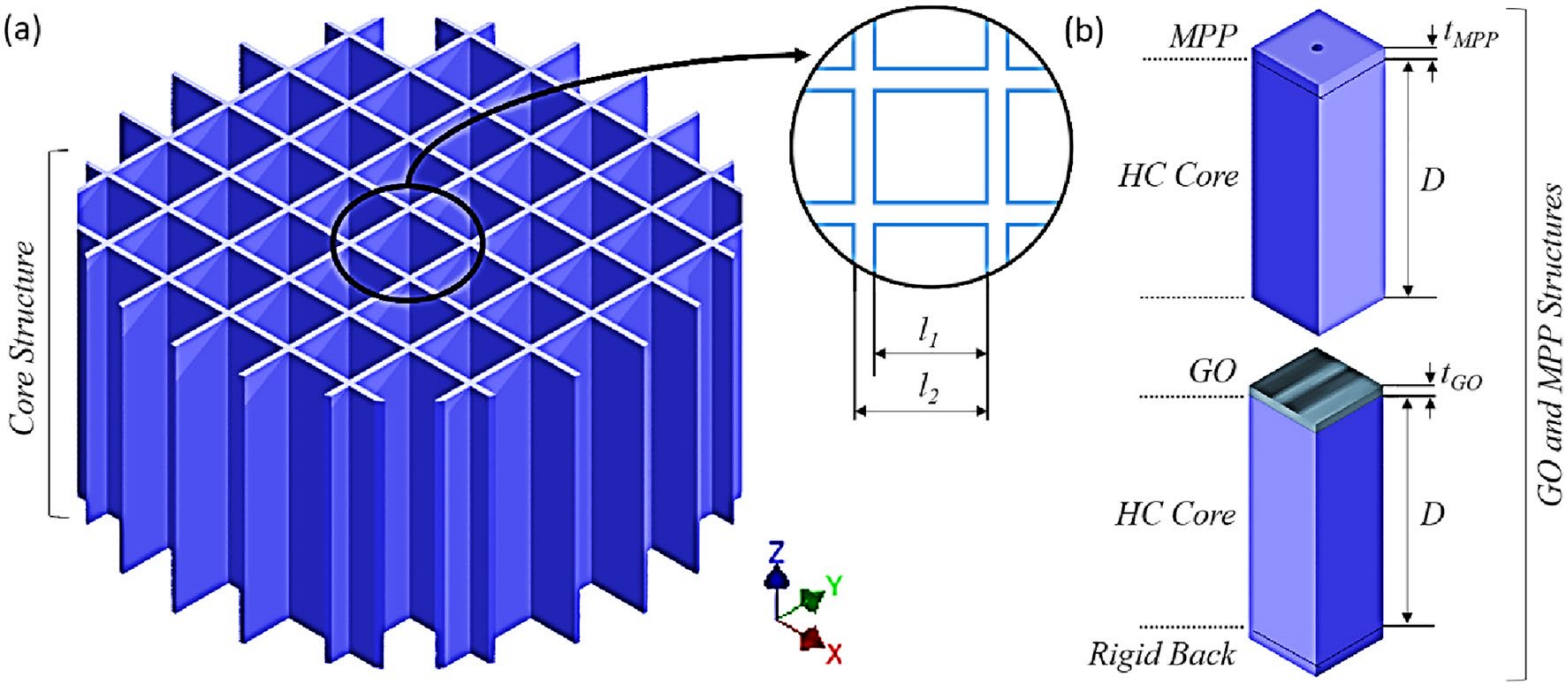
(**a**) Schematic of honeycomb (HC) structure which is either composed of a micro-perforated panel (MPP) as a top facesheet or a graphite oxide (GO) layer, (**b**) unit cell of MPP-HC and GO-HC. Sound absorption of the metamaterial is investigated from a plane acoustic wave normally incident to the top facesheet.

The proposed hybrid metamaterial is a periodic structure defined by its unit cell shown in Fig. 1. Based on 3D geometrical model of one unit cell, an analytical model is developed and experimental validated to describe the acoustic performances in terms of sound absorption for the different proposed configurations of the GO hybrid resonator metamaterial.

The normal sound absorption is calculated by the acoustic impedance definition of the single unit cell. Regarding the plate covered cavity configuration (GOHC structure), the acoustic impedance is estimated solving coupled acoustic-structural problem, while the MPP absorber configuration (MPPHC structure) is described by the equivalent electric circuit approach^36^.

The GOHC structure can be considered a plate covered cavity. A modal coupling theory based on fluid–structure interaction was used to calculate the coupled vibro-acoustic response. This information was then used to determine the absorption of the evaluated system. Membrane sound absorbers generally consist of rigid sides and back, covered with limp lightweight fronts and are used for low frequency absorption applications (but not limited to this). Predicting the response of these structures provides a quick tool to evaluate and design for specific acoustic conditions. In this case, the model was used to provide an analytical solution which was validated by the experimental results. The analytical model used in this work follows that outlined in^37^, where equations for determining the coupled structural–acoustic displacement response of a system are described^38^:

1 $$\left[ {\begin{array}{*{20}l} {\Lambda_{m} \left( {\omega_{m}^{2} - \omega^{2} } \right)} \hfill & { - S\left[ {C_{nm} } \right]^{T} } \hfill \\ {\left( { - \omega^{2} } \right)S\left[ {C_{nm} } \right]} \hfill & {\frac{{\Lambda_{n} }}{{\rho_{0} c_{0}^{2} }}\left( {\omega_{n}^{2} - \omega^{2} } \right)} \hfill \\ \end{array} } \right]\left[ {\frac{{w_{m} }}{{p_{n} }}} \right] = \left[ {\frac{{F_{m} }}{{Q_{n} }}} \right]$$

where: m and n are the structural and acoustic modes, ${w}_{m}$ is a vector of all structural modal participation factors, ${p}_{n}$ is a vector of all the acoustic modal participation factors. ${Q}_{n}$ is the modal volume acceleration, S is the total surface area of the structure in contact with the acoustic fluid, ${C}_{nm}$ is the dimensionless coupling coefficient, ${F}_{m}$ is the force acting on the structure, ${\Lambda }_{m}$ is the modal mass and ${\Lambda }_{n}$ the modal volume, $\omega$ is the structural displacement at a given frequency, ${\omega }_{m}$ structural modal participation factor, ${\omega }_{n}$ natural frequencies of the cavity, ${\rho }_{0}$ density of the fliud, ${c}_{0}$ speed of sound of sound in the fluid.

The acoustic impedance of the plate covered cavity (${Z}_{GO-HC}$) was calculated as follows:

2 $$Z_{GO - HC} = \frac{P}{{\rho_{0} c_{0} v}}$$

where $P$ is the external uniformly distributed sound pressure acting on the panel, $v$ velocity at depth of D.

The acoustic impedance of the MPPHC structure is calculated according with electric equivalent model proposed by Maa^36^, where the acoustic impedance for an MPP absorber is expressed as a series of the acoustic impedance related to the panel (*Z*_*MPP*_) and the acoustic impedance related to the enclosed cavity (Z_HC_). Moreover, the complex impedance for the MPP can be expressed in terms of real and imaginary part. The real part, named acoustic resistance (*R*_*MPP*_) represent the energy radiation and the viscous losses of the acoustic wave propagating through the perforations. The imaginary part, named acoustic reactance (*M*_*MPP*_) refers the mass of the air moving inside the perforation.

3 $$Z_{MPP} = \frac{{Z_{1} }}{{p\rho_{0} c}} = R_{1} - i\omega M_{1}$$

where *p* is the panel perforation ratio.

The acoustic impedance associated to the honeycomb core cell, which represents the enclosed cavity for the MPP absorber, depends on the mass of air behind the panel which is a function of the cavity depth,

4 $$Z_{HC} = i\;\cot \left( {Dk} \right)$$

where *k* the wavenumber.

Then the acoustic impedance for the MPP absorber is expressed as follow:

5 $$Z_{MPP - HC} = \left( {\frac{1}{{R_{1} - i\omega M_{1} }}} \right)^{ - 1} + Z_{HC}$$

The sound absorption of the hybrid structure, considering a normal incidence plane wave, can be calculated theoretically by determining the total acoustic impedance ($Z_{T}$) of the structure. The hybrid MPPHC and GOHC structures require two separate approaches in order to calculate the total acoustic impedance. The structure can be considered as a series–parallel connection system when considered in the format: MPPHCGOHC or GOHCGOHC ^39,40^. The absorption coefficient ($\alpha$)^41^ can then be calculated from the equations below:

6 $$\begin{aligned} & Z_{T} = Z_{MPP - HC} + Z_{GO - HC} \\ & Z_{T} = Z_{GO - HC} + Z_{GO - HC} \\ \end{aligned}$$

7 $$\alpha = \frac{{4{\text{Re}} \left( {Z_{T} } \right)}}{{\left( {1 + {\text{Re}} \left( {Z_{T} } \right)} \right)^{2} + \left( {1 + {\text{Im}} \left( {Z_{T} } \right)} \right)^{2} }}$$

## Results and comments

### Broadband sound absorption at low frequencies for proposed HGO

In order to achieve broadband sound absorption at low frequencies the influence of the thickness was investigated. Keeping constant the geometry of the HC unit cell (*l*_*1*_ and *l*_*2*_), the GO foil thickness and the geometrical characteristic of the MPP (panel thickness, perforation ratio and perforation diameters), the proposed hybrid structures behave different varying the HC core thickness. In particular, we compared the absorption properties of hybrid structures with three different global thickness 30 mm, 50 mm and 70 mm for GOHCGOHC and MPPHCGOHC structures with the design parameters summarized in Tables 1 and 2.

**Table 1:** Design parameters of proposed GOHCGOHC with different HC thickness.

Sample	Structure	GO foil thickness (μm)	HC core thickness (mm)	Total thickness (mm)
GOHCGOHC-30	GO foil + HC + GO foil + HC	30	15	30
GOHCGOHC-50	GO foil + HC + GO foil + HC	30	25	50
GOHCGOHC-70	GO foil + HC + GO foil + HC	30	35	70

**Table 2:** Design parameters of proposed GOHCGOHC and MPPHCGOHC structures with different HC thickness.

Sample	Structure	GO foil thickness (μm)	HC core thickness (mm)	MPP thickness (mm)	MPP perforation ratio (%)	Total thickness (mm)
GOHCGOHC-30	GO foil + HC + GO foil + HC	30	15			30
MPPHCGOHC-30	MPP + HC core + GO foil + HC core	30	15	1.5	6.0	30
GOHCGOHC-50	GO foil + HC + GO foil + HC	30	25			50
MPPHCGOHC-50	MPP + HC core + GO foil + HC core	30	25	1.5	6.0	50
GOHCGOHC-70	GO foil + HC + GO foil + HC	30	35			70
MPPHCGOHC-70	MPP + HC core + GO foil + HC core	30	35	1.5	6.0	70

**Figure 2:**
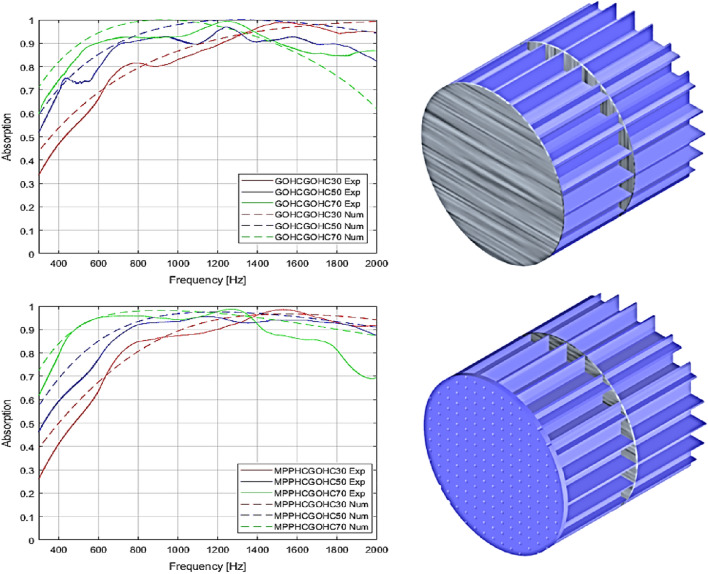
Absorption coefficient for the proposed MPPHCGOHC and GOHCGOHC structures with different thickness compared with the analytical results.

A good agreement between the experimental results and the analytical ones is shown in Fig. 2 except for the boundary frequency regions of the plot where the discrepancies are mainly due to the limit working frequency range of the experimental test rig. A broadband sound absorption is achieved with all the different thickness configurations. In particular 15 mm thickness of the single HC core and 30 mm global structure thickness allows a broadband absorption from 550 Hz with global sound absorption over 50% and over 90% from 1400 Hz. A 50 mm global thickness result the best configuration in terms of broadening sound absorption with absorption properties over 50% from 300 Hz and over 90% from 700 Hz. Increasing the global thickness to 70 mm, the maximum absorption is moving at lower frequencies but with a reduction of the broadening properties compared with the 50 mm thickness. In this case we can guarantee a sound absorption over 90% from 400 Hz but we reduce the capability of sound absorption over 1500 Hz. Moreover, we demonstrate how, keeping constant the thickness and all the other geometrical parameters, using an MPP with high perforation ratio as external skin for the hybrid resonator the same sound absorption properties are guaranteed compared with a GO foil as external skin. The discrepancies between the numerical and measurement results for both MPPHCGOHC and GOHCGOHC are mainly related to small geometrical errors of the 3D printed parts (so the HC core structures) like for example small error on the millimeter wall thickness of the HC cell. Furthermore, errors occur relating to the size of the circumferential cells. In the numerical model, a square unit cell is considered and the absorption properties are estimated solving the series–parallel arrangement of n-cells with identical geometrical size. However, the tested prototype has a circular shape to fit into the testing apparatus (impedance tube) which means the circumferential cells are not perfectly square. So there were a small discrepancy in terms of size of the circumferential cells between the numerical model and the tested prototype. Moreover, the circumferential edge of the embedded GO foil is free because of the circular shape of the sample, while in the numerical model all the edge of the square unit cell are assumed clamped. Such difference on the boundary conditions on the circumferential edge can justified the small mismatched between numerical and experimental results of Fig. 2.

**Figure 3:**
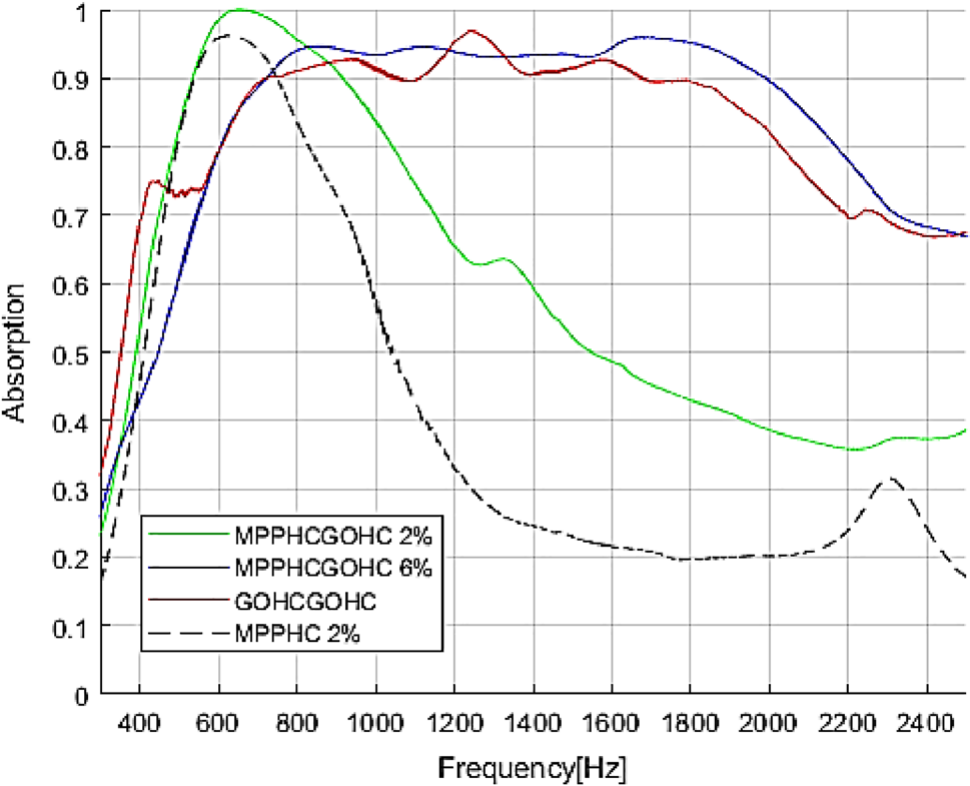
Comparison of GOHCGOHC structure with the MPPHCGOHC structure with different perforation ratio (2% and 6%) and the MPPHC structure. The global thickness of the structures is 50 mm.

To investigate the effect of the perforation ratio on the sound absorption properties, two different perforation ratios were compared in Fig. 3. In particular the MPPGOHCGO structure with 2% and 6% of MPP perforation ratio are compared with equivalent GOHCGOHC with 50 mm thickness.

As shown in Fig. 3 the sound absorption properties of the MPPGOHCGO structure is strictly related to the entity of perforation because depending on the perforation ratio the effect of GO embedded foil will be considerable or not. For small grade of perforation, the absorption profile shows a unitary peak at 620 Hz which is due to the main resonance of the preceding MPP absorber. In fact comparing the absorption profile of the MPPHCGOHC and the equivalent MPPHC structure with same perforation ratio, we can identify the same trend with single absorption peaks at same frequency 620 Hz. In other words, the effect of the GO embedded foil is negligible for low perforation ratio. So for the MPPHCGOHC structure with 2% of perforation, the GO induce a small contribution to increase the absorption level at higher frequencies, but the absorption properties are mainly dominated by the preceding MPP structure because the dynamic of the GO is not excited by the incoming sound wave due to the low porosity. However, increasing the perforation ratio to 6%, there is a complete superposition principle, so the net absorption performances of MPPHCHOHC structure is the combination result between the low frequency absorption due the resonance plus the viscous loss of the preceding MPP and the broadening effect associated to the embedded GO foil.

To demonstrate the advantages induced by the GO on the absorption performances, the proposed GOHCGOHC and MPPHCGOHC are compared with equivalent commonly used MPP absorber backed by air cavity and MPP backed by HC core. The compared structures, reported in Table 3, are tested keeping constant the global thickness of the structure 50 mm. The compared structures present the same HC core unit cell geometry and the same geometrical parameter for the MPP.

**Table 3:** Design parameters for the proposed metamaterial structure and the equivalent commonly used absorber based on MPP. The global thickness for all the tested structure is 50 mm.

Sample	Structure	GO foil thickness (μm)	HC core thickness (mm)	MPP thickness (mm)	MPP perforation ratio (%)	Air-gap (mm)
GOHCGOHC-50	GO foil + HC + GO foil + HC	30	25			
MPPHCGOHC-50	MPP + HC core + GO Foil + HC core	30	25	1.5	6.0	
MPP	MPP + air-gap			1.5	6.0	50
MPPHC	MPP + HC core		50	1.5	6.0	
MPPHCMPPHC	MPP + HC core + MPP + HC core		25	1.5	6.0	

**Figure 4:**
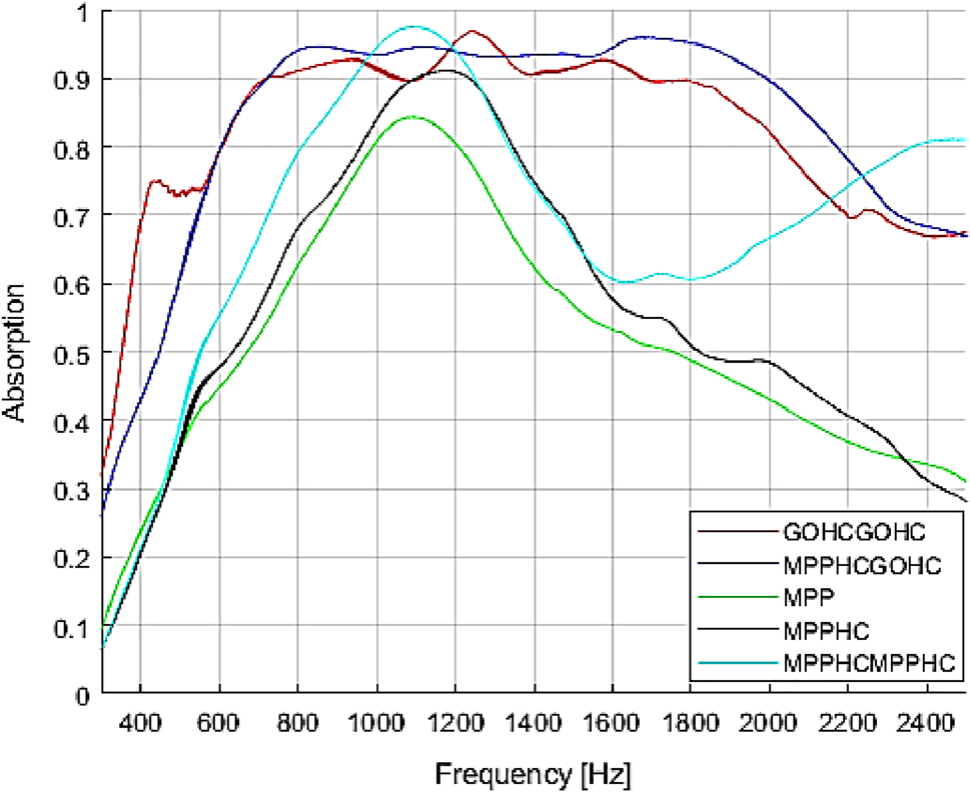
Comparison between the proposed metamaterial structure GOHCGOHC and MPPHCGPHC, the MPP absorber, the MPPHC structure and MPPHCMPPHC structure. All the tested structure present the same global thickness (50mmm) and the same geometrical parameter (MPP thickness 1.5 mm, perforation ratio 6% GO foil thickness 30 μm).

In Fig. 4 are plotted the absorption coefficient measured for the 5 tested structures. As expected the MPP absorber realized by micro perforated panel with submillimetre perforation comparable with the boundary layer thickness resonant enclosed air cavity, is characterized by a single absorption peak around the resonance frequency $f=\frac{c}{2\pi }\sqrt{\frac{S}{VL}}$ ^42^, where *c* is the speed of sound, *S* is the perforation area and *V* is the cavity volume. In fact, the MPP absorber behave as a mass spring single degree of freedom system where the mass is the mass moving into the perforation and the stiffness is related to volume of air in the resonant cavity, so the absorption present only one peaks related to the system resonance around 1100 Hz. The designed honeycomb core does not affect the absorption performances of the MPP in terms of broadening or shifting in frequency the absorption peak. Because of the regularity of the core size, no fluctuation or changes in the perforation ratio between the core unit cells could affect the sound absorption. As a result the single core cell of the MPPHC structure acts as a single MPP absorber with the same perforation ratio and an equivalent cavity volume, which is characterized by the same resonance frequency. However a small enhancement in the absorption level is measured due to the contribution of all the core cell to the sound absorption. Considering now the MPPHCMPPHC where the embedded Go foil is replaced by a second MPP. As demonstrated in previous work^43^, the effect on the sound absorption of a MPP series is to sum the single MPP contribution. Then the absorption profile is characterized by two main absorption peaks, the first one at same frequency of the MPPHC structure associated to the first MPP backed by 50 mm thickness of acoustic resonant volume, and the second one related to second MPP which is backed by a smaller resonator acoustic volume (25 mm).

Therefore the proposed GOHCGOHC and MPPHCGOHC metamaterial resonator show the best low-frequency and broadband sound absorption properties. Such metamaterials represent a powerful subwavelength $\frac{1}{14}\lambda$ solution for broadband sound absorption at low frequencies.

### GO based metamaterial as multi degree of freedom hybrid resonator

In Fig. 2 the proposed GOHCGOHC structure metamaterial show that the unit cell of the proposed metamaterial does not behave as a single degree of freedom resonator, as the common MPP absorbers of the membrane-type metamaterial, but as multi degree of freedom resonator and the absorption broadening characteristics are then associated to multiple hybrid resonances. We demonstrated in a previous work^44^ how considering the fluid–structure interaction of thin plate with the enclosed airgap, the plate does not behave as a piston system as the other membrane-type acoustic metamaterials^10,17,20^. Considering the single HC core unit cell, it acts as a senator acoustic cavity covered by the GO foil behaving as a plate fixed supported on the HC cell edges. There is a mutual interaction between the GO foil and the HC core cavity. The vibration of GO foil is perturbed by the fluid pressure loading and the acoustic filed in the cavity is influenced by the dynamic response of the foil itself. In particular, the non-uniform pressure distribution due to higher acoustic modes of the HC core cavity will act on the GO foil which, on the other hand will react according with its intrinsic mode shapes related to its structural resonances excited by the incoming sound wave excitation. This interaction between the higher acoustic modes and structural modes generated multiple hybrid resonances which dissipate the sound energy associated to the incoming sound wave. As a consequence, the multiple hybrid resonances generated from the interaction between the high orders vibration modes of the GO foil and the non-uniform pressure distribution on the HC cavity guarantees a broadband sound absorption. To achieve a higher absorption at lower frequencies two GOHC structures are stuck together forming the GOHCGOHC structure. In this case, the interaction between the high order structural modes of the GO foil and the higher acoustic modes of the cavity are still on introducing multiple hybrid resonances and ensuring a broadband absorption. Moreover, for the GOHCGOHC the first GO layer see a global acoustic volume of 50 mm thickness due to the two stuck HC core which means moving a lower frequencies the acoustic mode of the HC cavity.

**Figure 5:**
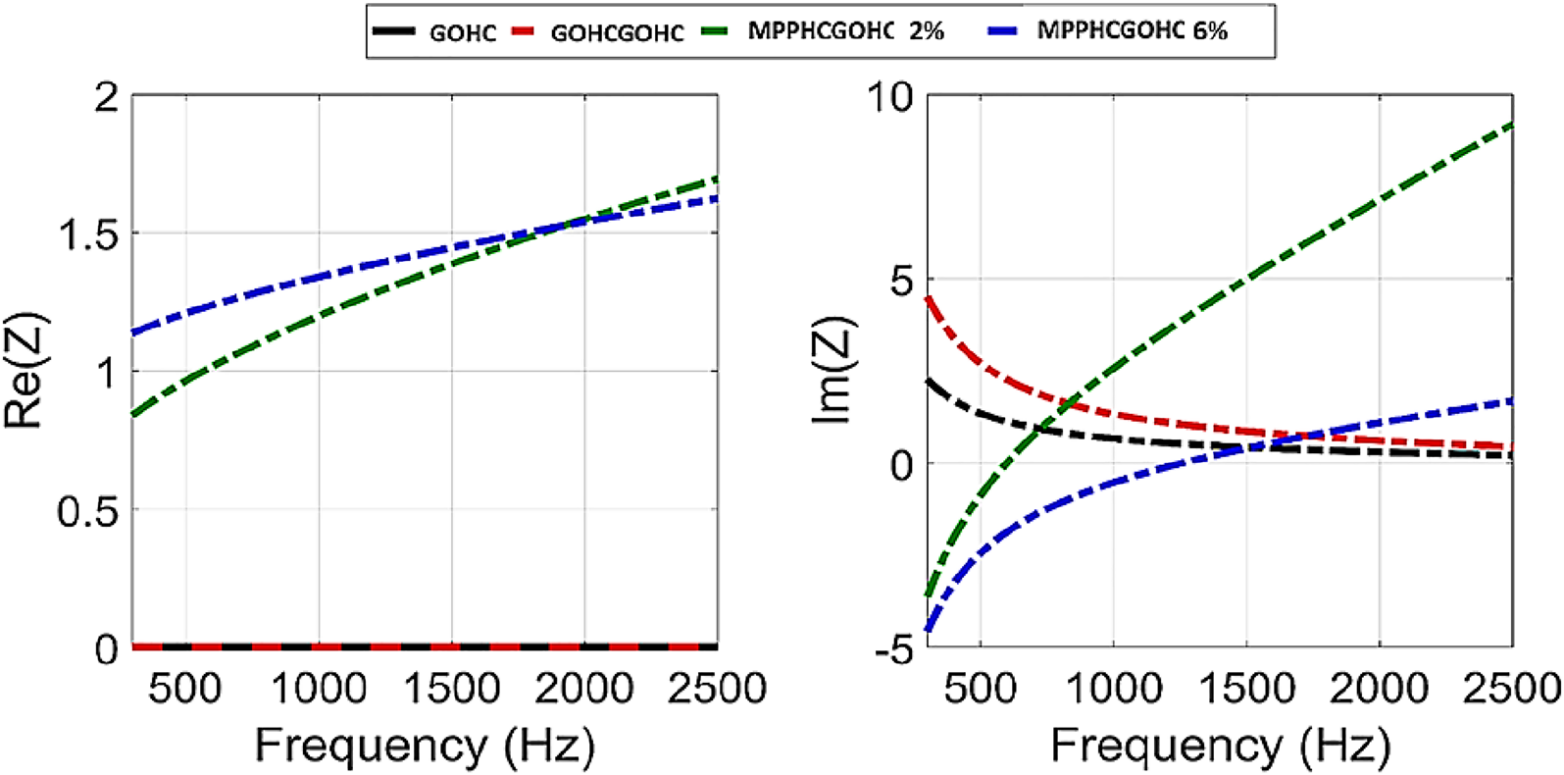
Acoustic Reactance (Im(Z_T_)) and Acoustic Resistance (Re(Z_T_)) for the proposed Hybrid metamaterial structures: GOHC, GOHCGOHC, MPPHCGOHC with 2% of perforation ratio and MPPHCGOHC with 6% of perforation ratio.

The absorption performances of metamaterial are usually strictly related to the acoustic impedance and acoustic reactance in order to achieve an impedance matching. The class of resonator metamaterial in particular are characterize by zero acoustic reactance (*Im(Z*_*T*_*)* = *0*) which indicates the resonance frequency of the acoustic chamber and unitary acoustic resistance (*Re(Z*_*T*_*)* = *1*) which indicates impedance matching with the impedance of air^24,30^. In Fig. 5 we plot the acoustic reactance and the acoustic resistance for the proposed hybrid metamaterial structure. Starting from the MPPHCGOHC with 2% of perforation ratio structure (green line), the acoustic resistance cross 1 at 600 Hz and at same frequency the acoustic reactance is zero. The combination of zero reactance and unitary resistance confirms that for this structure the GO effect is negligible and the high absorption at 600 Hz is due to the acoustic resonance of the HC core cell (Im(Z_T_) = 0) and impedance matching (Re(Z_T_) = 1) due to the MPP as for other resonator metamaterials. Increasing the perforation ratio, the effect of the GO becomes important on the absorption characteristic. In fact for the MPPHCGOHC structure with 6% of perforation ratio, the reactance is zero around 1600 Hz which the frequency where the preceding MPPHC structure presents the main acoustic resonance of the HC core cell but the acoustic resistance is never equal 1 in the frequency range of interest. Together with GOHCGOHC structure is analysed also the single GOHC structure which show and completely different behaviour compared with the usual resonator metamaterials. The acoustic resistance is never assume unitary value but is constant around zero which means that the great absorption properties of this structure are not related to impedance matching with the incoming sound wave. However, the acoustic reactance is always positive and assume an asymptotic behaviour to zero but it is never zero in the frequency range of interest. This confirm the hypothesis that the broadband absorption for the GOHC structures is associated to the multiple hybrid resonances not only associated to the acoustic resonance of the HC cavity but to the mutual interaction between the structural dynamic response for the GO foil and the higher acoustic modes of the HC cell, as discussed above. The better acoustic performances is also confirmed from the acoustic reactance plot, where moving from GOHC to GOHCGOHC structure the reactance is more important because it has a larger quantity in the low frequency range.

## Discussions

Recently introduced and studied acoustic metamaterials, such as membrane-type metamaterials, Helmholtz’s resonator-type metamaterials and MPP-type metamaterials allow good sound absorption properties at low frequencies, but broadband sound absorption at low frequencies with relative thin structure is still challenging. In this report, we proposed a novel acoustic Hybrid Multi-Degree of Freedom resonator Metamaterial based on a lightweight honeycomb core structure (HC) with skin and embedded submillimetre Graphite Oxide (GO) foils. Two main configurations are proposed; GOHCGOHC and MPPHCGOHC, which provide broadband high sound absorption while maintaining excellent mechanical stiffness/strength properties related to the HC core structure. Making use of the electro-acoustical equivalent circuit theory for the MPP and the coupled acoustic-structural motion equation for the GO and HC structure, we developed an analytical model to compute the absorption properties through the evaluation and combination of the acoustic impedance. The analytical and experimental results show good agreement, demonstrating that the proposed metamaterial with GOHCGOHC and MPPHCGOHC structures achieve a nearly perfect broadband absorption at low frequencies. We demonstrated how the broadband characteristic is mainly due to multiple hybrid structural–acoustic resonances that arise because of mutual interaction between the high order structural and acoustic vibration modes. The proposed Hybrid GO based metamaterial with a structure of 50 mm thickness represents a subwavelength acoustic metamaterial with outstanding nearly perfect absorption over a broadband low frequency range from 300 to 2500 Hz. In Table 4 the sound absorption property for the proposed GOHCGOHC-MPPHCGOHC metamaterials with other relevant acoustic metamaterials-absorber.

**Table 4:** Comparison of sound absorption characteristic for the proposed GOHCGOHC-MPPHCGOHC and other relevant acoustic metamaterial-absorber.

Metamaterial type	Absorption level	Frequency range (Hz)	Thickness (mm)
Membrane-type^16^	Over 60%	190 Hz—single peak	56
Membrane-type^17^	Over 60%	250 Hz—single peak	30
Single MPP	Over 60%	620 Hz—single peak	50
MPP multilayers^43^	Over 60%	500–2700 Hz broadband	66
Hybrid MPP-coil up channel^28^	Over 60%	300–550 Hz broadband	50
Hybrid MPP-perforated honeycomb corrugation^30^	Over 60%	350–1000 Hz broadband	60
MPPHCGOHC—GOHCGOHC	Over 60%	350–2000 Hz broadband	50

## Methods

### GO hybrid metamaterial manufacturing

The proposed metamaterial consisted of various combinations of MPP-HC and GO-HC as outlined in Fig. 1. The HC core structure was 3d printed using stereolithographic technology using Formlabs Tough Resin (refer to Method for detail) which has good mechanical stiffness and strength, thus it is considered acoustically rigid in the theoretical model. An aluminium facesheet was used as the backing of the structure when tested in an Impedance Tube test rig. The HC core has a square cross-section with an inner side length l_1_ (6 mm) and a unit cell side length l_2_ (6.5 mm, refer to Fig. 1a). The thickness of the MPP and GO top facesheets are t_MPP_ (t_MPP_ = 1.5 mm) and t_GO_ (t_GO_ = 30 μm). The depth of the HC core is D (25 mm, if not otherwise specified), with multiple cores stacked to increase this depth. This design of the metamaterial combines the effects of the MPP resonator with that of a membrane covered cavity.

**Figure 6:**
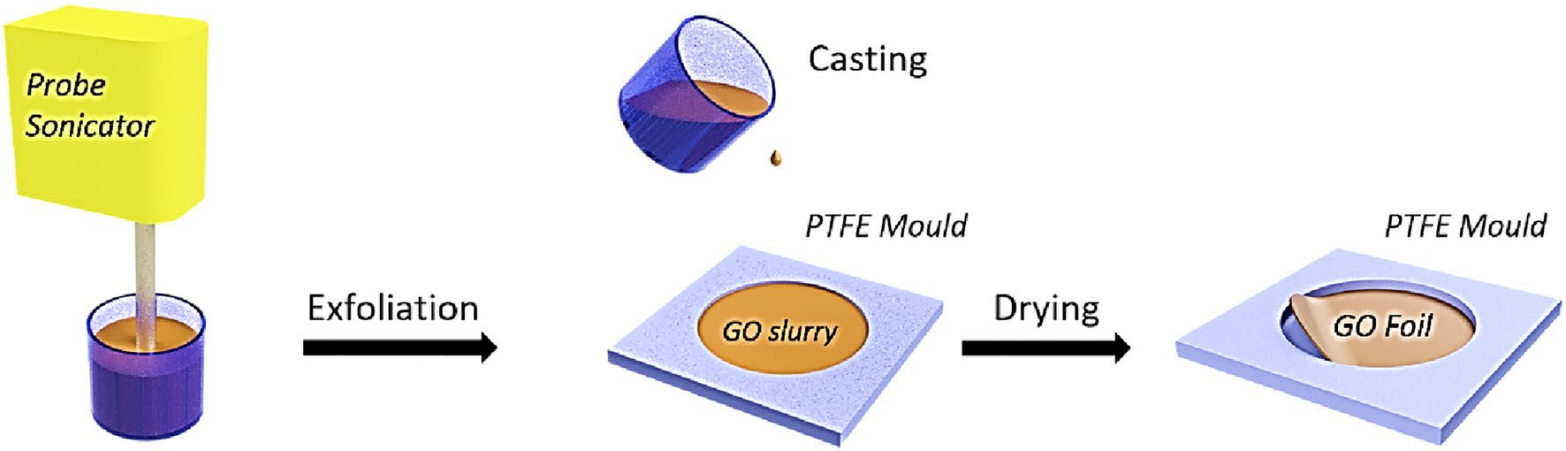
Manufacturing process scheme of GO micro-sheets.

The GO micro-sheets were manufactured starting from Graphite Oxide (GtO) powder through sonication treatments (Fig. 6). GtO powder (TOB-2430, Xiamen TOB New Energy Co., LTD) was added to distilled water and gently stirred for 30 min to obtain a dispersion with a concentration of 8 mg ml^−1^. The GtO dispersion was then exfoliated to GO thanks to the mechanical excitation provided by a probe sonicator (UP100H, Dr. Hielscher GmbH) for 1 h with 100% amplitude and continuous power discharge, under an ice bath to prevent the temperature to raise and with gentle stirring to ensure a homogeneous exfoliation process. The obtained slurry was then casted in PTFE moulds, exploiting its low surface free energy for an easy release of the foils, with a circular shape of 8 cm diameter. The thickness of each sample was controlled by adjusting the volume of the GO suspension poured inside the mould. The GO Foils were finally obtained, peeling them off the PTFE moulds after 24 h of oven drying at 40 °C, and cut to the desired size.

### Graphite-oxide characterization

First microscopic characterization of GO foil was performed through scanning electron microscopy (SEM) analysis and X-Ray diffraction analysis.

**Figure 7:**
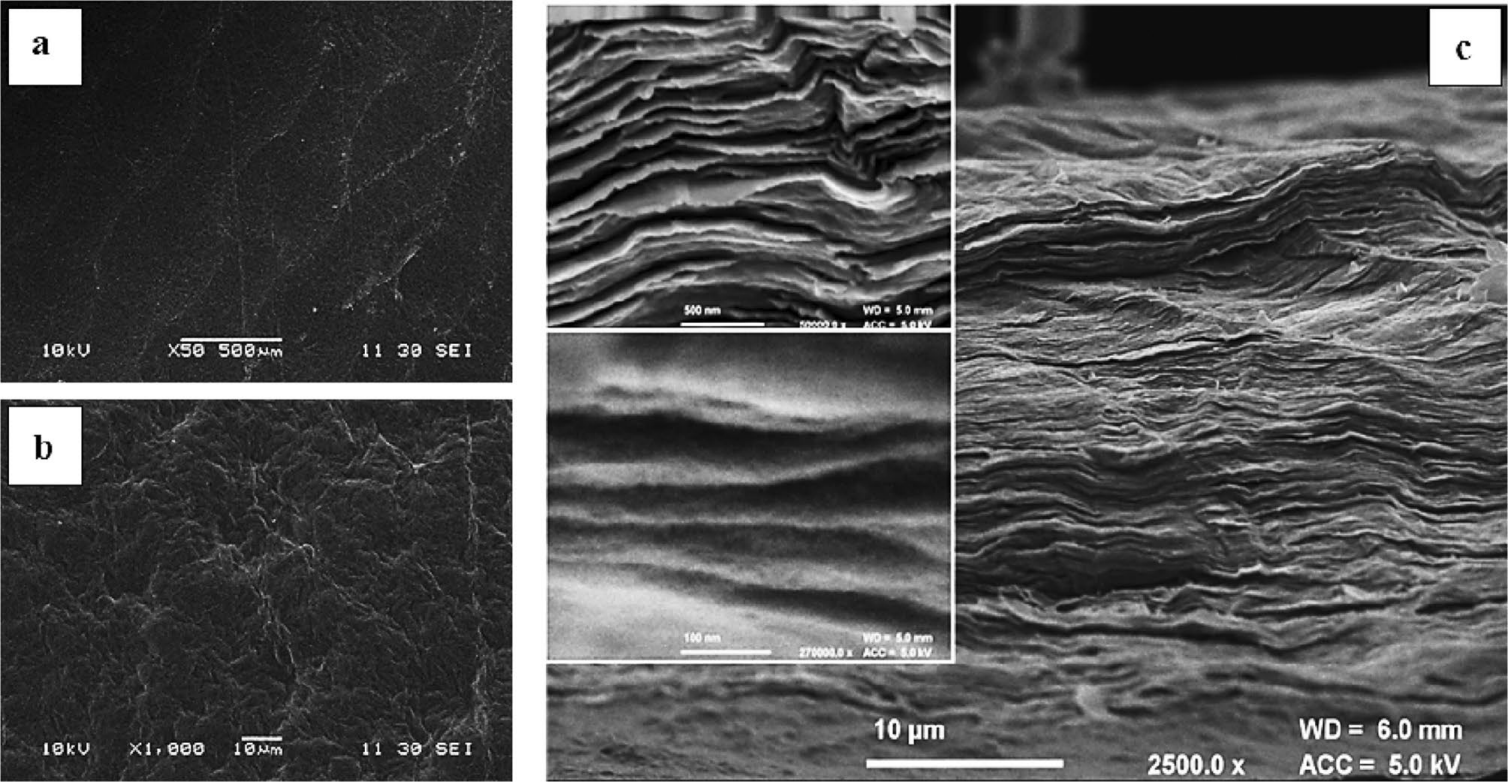
(**a**) Low-magnification SEM image of GO foil. (**b**) High magnification (×10,000) SEM image of GO foil. (**c**) Cross section SEM image of GO foil with onset at higher magnification (top100k, bottom 270 k).

The surface of a GO Foil specimen imaged via scanning electron microscopy (SEM) shows a distinctive chain-like pattern in the low magnification image, attributable to the stress induced by the surface tension between the liquid suspension and the sides of the mould during the drying process (Fig. 7a). In the high magnification image, it’s worth noting that during the self-assembly of the foil a wrinkled morphology is obtained (Fig. 7b), made by the non-ordered overlapping of GO layers. The cross-section images of a cryogenically fractured GO Foil confirmed the non-ordered overlapping of GO layers and revealed a nanolayered structure where each layer, having a thickness of few nanometres, is well distinguishable (Fig. 7c).

**Figure 8:**
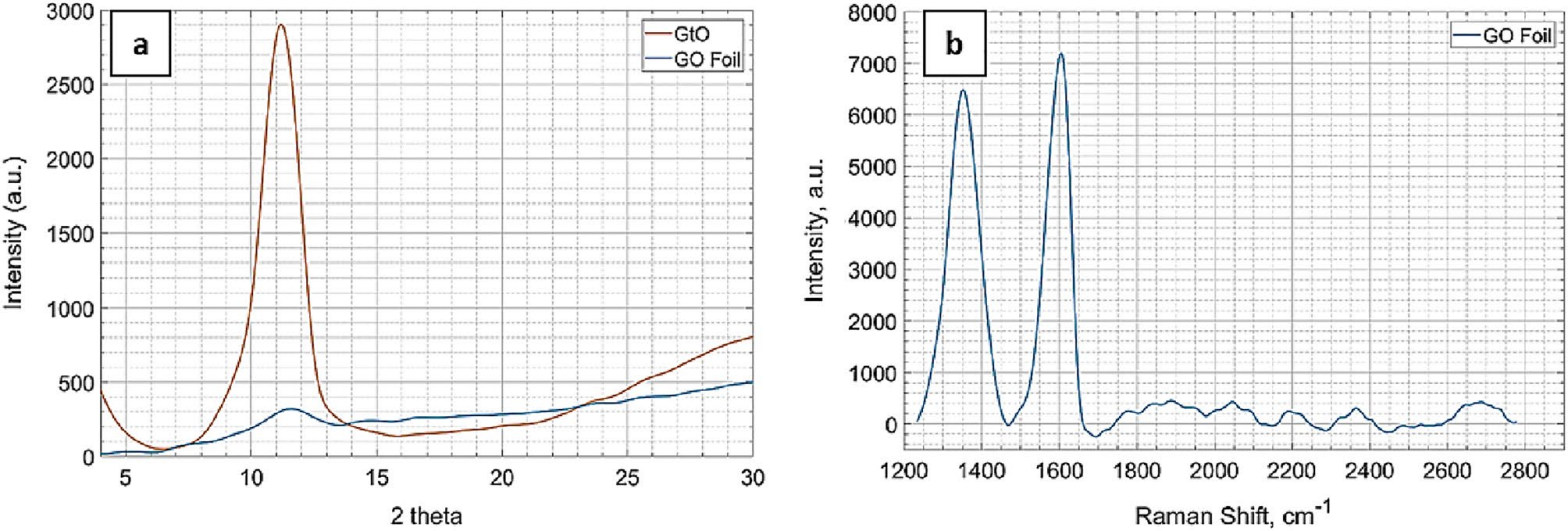
(**a**) XRD patterns of GtO powder and GO Foil. (**b**) Raman spectra of the GO Foil.

The X-Ray diffraction patterns of the pristine GtO powder and GO Foil are shown in Fig. 8a. The (001) reflection peak, typical in GtO and useful to characterize the interplanar spacing (d-spacing) between the oxidised graphene layers^45^, is found at 2θ = 11.20° with its amplitude strongly reduced and its position slightly shifted to 2θ = 11.59° after the exfoliation and casting process. The application of Bragg’s law allowed the calculation of d-spacings in in GtO powder and GO Foil, with results of respectively d = 7.89 Å and d = 7.63 Å. These findings confirm a successful exfoliation of GtO in GO and that no restacking happened during the foil manufacturing process, in accordance with Kashayap et al. work^46^.

The Raman spectra in Fig. 8b presents the typical D and G bands of carbon materials at respectively 1352 cm^−1^ and 1604 cm^−1^. The first arises from breathing modes of sp^2^ atoms in rings while the second is characteristic of primary in plane vibrations mode, their intensity ratio (D/G ratio) can be used to describe the order of the system: in single layer graphene it approaches zero while higher values are associated to more disordered structures^47^. A D/G ratio of 0.88 was found for the GO Foil, confirming the existence of structural defects introduced after the oxidation of Graphite.

**Figure 9:**
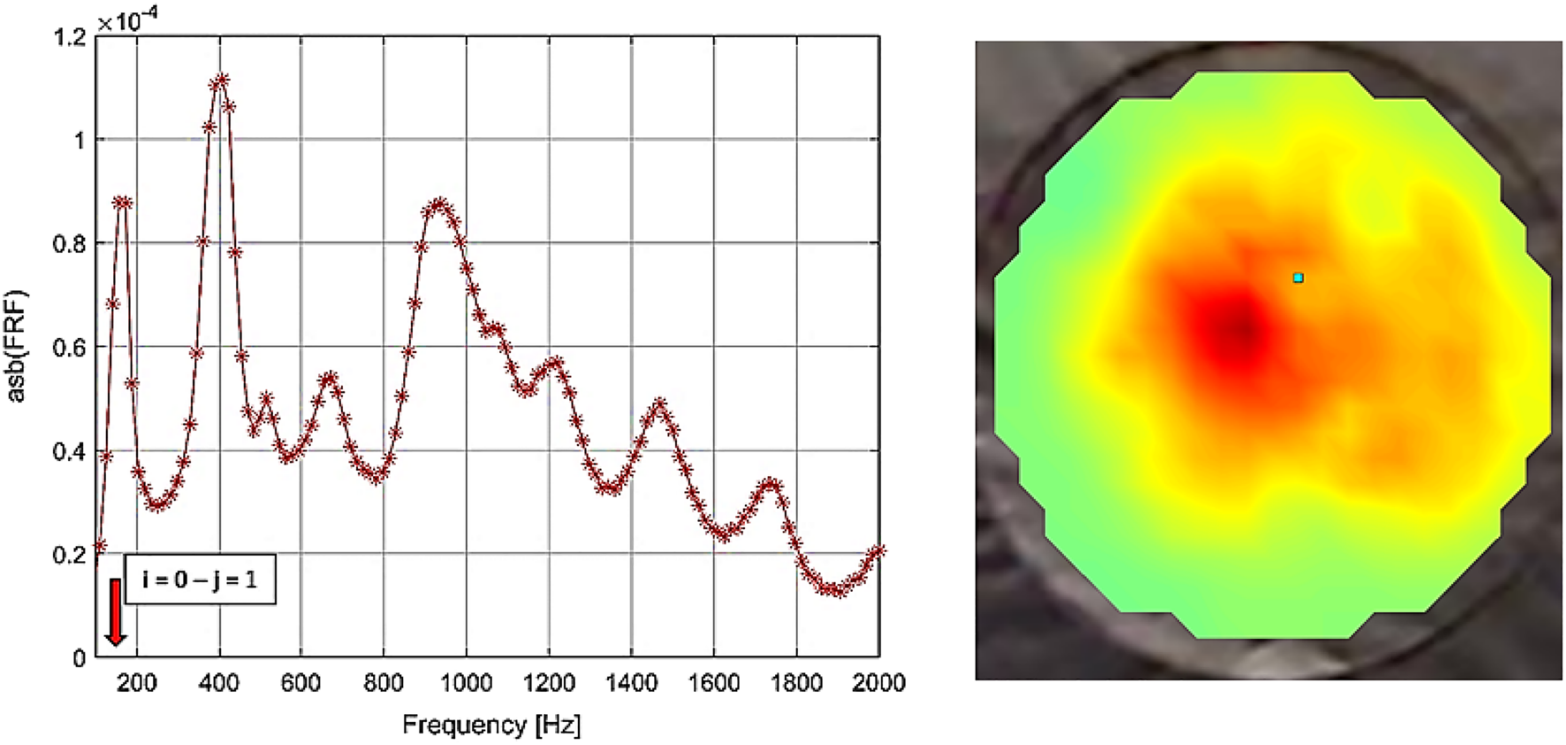
Frequency response function of graphene-oxide foil (h = 35 µm, a = 0.0254 m, ρ = 1800 kg/m^3^), and mode shape (i = 0, j = 1) measured by laser-vibrometer rig.

Macroscopic characterization was also performed to investigate the elastic properties of the GO foil. In particular the Elastic Modulus of manufactured GO foil was measured using a non-destructive approach based on the resonances of the foil (Fig. 9). The used non-destructive approach is based on the plate’s theory. According with^6^ the natural frequencies are function of the indices associated with the number of flexural half-waves in the two plate dimensions. Moreover, they are related to the material properties following the equation

8 $$f_{ij} = \frac{{\lambda_{IJ}^{2} }}{{2\pi a^{2} }}\left[ {\frac{{Eh^{3} }}{{12\gamma \left( {1 - \nu^{2} } \right)^{{}} }}} \right]^{2} \quad \left\{ {\begin{array}{*{20}c} {i = 1,2,3, \ldots } \\ {j = 1,2,3, \ldots } \\ \end{array} } \right.$$

where ${\lambda }_{ij}$ is a dimensionless which is function of the mode indices, *a* is the characteristic dimension of the plate, *h* is the plate thickness, *ν* is the Poisson’s ration, *E* the Young modulus and *γ* is the mass per unit area of the plate. So the Eq. (8) can be solved in term of E when the i–j-th natural frequency is known. So the GO foil of 35 µm thickness (*h*) and 0.0254 m radius (*a*) is placed in one end of an impedance tube and a plane wave generated into the tube is used to excite the foil. The structural dynamic response of the plate is measured focusing a laser-vibrometer on a grid of points on the downstream surface of the foil. Since a plane wave can excite only the symmetric bending modes of the foil, from the measured frequency response function, the frequencies of i–j-th bending mode can be estimated.

**Figure 10:**
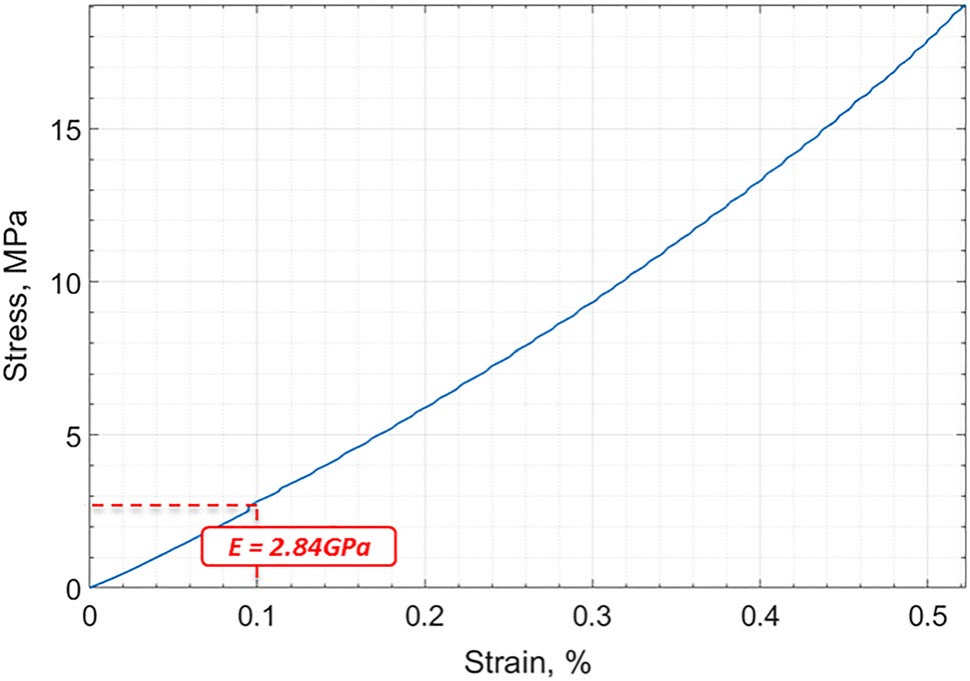
Stress–strain curve for GO foil (20 mm × 5 mm and 35 µm thickness) measured by DMA.

Considering a circular plate under clamped edges boundary conditions and normal distributed load, the dimensionless parameter ${\lambda }_{01}=39.77$ for i = 0 and j = 1 (according with^6^), and the natural frequency for such mode is 160 Hz (according with Eq. (8)). So for the proposed GO foil the elastic modulus is E = 2.9 e + 09 Pa. This result was confirmed by Dynamic Mechanical Analysis (DMA, Tritec 2000 DMA, Tryton Technology Ltd) in tension mode with ramp loading of 0.02 N min^−1^ applied on GO specimen cut in stripes (20 mm × 5 mm). The Elastic Modulus extracted from the measured stress–strain curve was 2.84 GPa (Fig. 10).

### Acoustic properties measurement test rig

The acoustic performance in terms of normal sound absorption was characterized with an in-house impedance tube test rig, consisting of an aluminium impedance tube with circular cross section and internal dimeter of 50.8 mm, a full range speaker (2″, BMS 4592 Compression Driver), pressure-field microphones (1/4″, 10 mV/Pa, GRAS 40PL-CCP Free-field Array Microphones), audio power amplifier (model: Behringer: NU1000, High-Density 1000-W Power Amplifier), data acquisition device (24-bit, 105.4 kS/s/ch, model: DT9837C Dynamic Signal Analyser, Data Translation)**.** The standardized Transfer Function Method was used to measure the normal sound absorption coefficient staring from the standing wave pressure measured at two microphone stations. The measured pressure signals were processed with LabView user interface implemented in the test rig.

Honeycomb structures were printed with Formlabs Form 3^48^ (XY resolution 25 μm, layer thickness 25 μm, 250 mW Laser Power) using Formlabs Tough Resin^49^.

## Data Availability

The datasets used and/or analysed during the current study available from the corresponding author on reasonable request.
